# Eosinophilic cholangitis misdiagnosed as cholangiocarcinoma: a case report

**DOI:** 10.3389/fonc.2026.1791076

**Published:** 2026-03-17

**Authors:** Yabo Hou, Xinxin Wang, Jianhua Chang, Xiaojun Yang

**Affiliations:** Department of Hepatobiliary Surgery, Gansu Provincial Hospital, Lanzhou, Gansu, China

**Keywords:** eosinophilia, eosinophilic cholangitis, obstructive jaundice, pathological examination, sclerosing cholangitis

## Abstract

Eosinophilic cholangitis (EC) has a low clinical incidence, and cases with definitive pathological confirmation are extremely rare. Due to the lack of specificity in clinical and radiologic features, EC is frequently misdiagnosed as cholangiocarcinoma. We report a 65-year-old man admitted with progressive jaundice and persistent dull right upper-quadrant abdominal pain for more than 1 month. Laboratory tests showed a markedly elevated peripheral blood eosinophil count, significantly elevated bilirubin levels, and elevated tumor markers. MRI revealed intra- and extrahepatic bile duct dilatation and common bile duct wall thickening. Cholangiocarcinoma was initially suspected. A radical pancreaticoduodenectomy was planned. However, intraoperative exploration showed edema and induration of the bile duct and pancreas. Therefore, the radical resection was aborted, and bile duct wall biopsy alone was performed for pathological diagnosis. Postoperative histopathology revealed dense eosinophilic infiltration in the bile duct wall (>20 eosinophils per high-power field), confirming the diagnosis of EC. After postoperative standard methylprednisolone therapy, the patient achieved complete symptom resolution. Laboratory indices returned to the normal range, and no recurrence was observed during the 3-month follow-up. This case highlights that EC should be suspected in biliary obstruction with unexplained eosinophilia, and histopathology is the key to definitive diagnosis.

## 1 Introduction

Eosinophilic cholangitis (EC) is a rare inflammatory biliary tract disorder characterized by eosinophilic infiltration of the bile duct wall and peripheral eosinophilia (1). EC is frequently accompanied by allergic disorders or parasitic infections (2). Clinical symptoms (e.g., jaundice and abdominal pain) and radiologic findings (e.g., bile duct dilatation and ductal wall thickening) in EC substantially overlap with those of primary sclerosing cholangitis (PSC), IgG4-related sclerosing cholangitis (IgG4-SC), and cholangiocarcinoma (CCA), resulting in a high rate of clinical misdiagnosis. Curative-intent radical resection is the primary therapy for cholangiocarcinoma, whereas glucocorticoids are the first-line treatment for EC. Misdiagnosis may result in overtreatment and unnecessary medical interventions, increasing the physical, psychological, and economic burden on patients. To help address the aforementioned diagnostic challenges, this study presents a detailed analysis of a confirmed EC case and discusses key diagnostic, therapeutic, and differential considerations, aiming to improve clinical diagnostic accuracy and enhance disease recognition.

## 2 Case report

**Figure 1:**
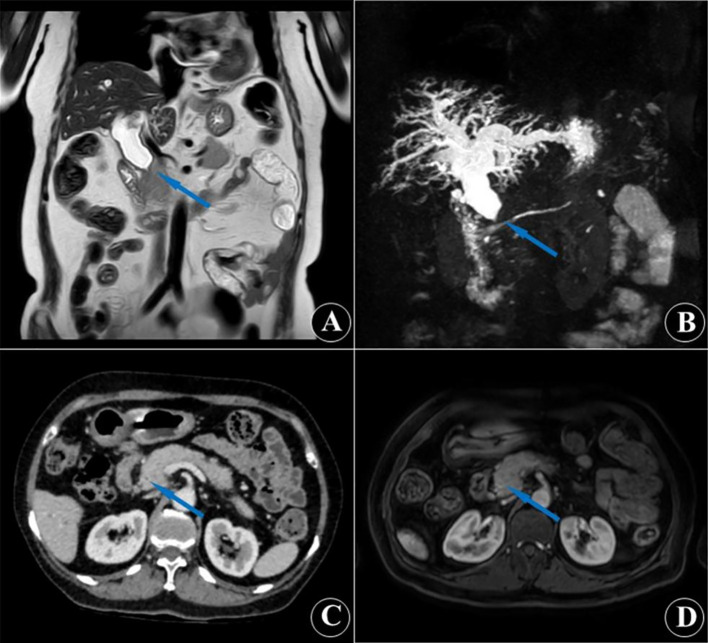
Contrast-enhanced MRI **(A, D)**, MRCP **(B)** and contrast-enhanced CT **(C)** showing features of biliary obstruction, including wall thickening and luminal narrowing of the distal common bile duct (blue arrow).

A 65-year-old man was admitted for progressive jaundice of the skin and sclera for more than 1 month. He denied chills, fever, diarrhea, alcohol use, any history of allergic disease, or hepatotoxic/special drug exposure. Laboratory evaluation on admission showed that viral hepatitis markers, serum IgG4, and autoimmune hepatitis–related autoantibodies were negative. Serum CA19–9 was elevated at 217 U/L. Stool ova and parasite (O&P) examination was negative, ruling out parasitic infection; Serum total IgE level was 38.5 IU/mL (reference range 0–125 IU/mL). Contrast-enhanced MRI (with MRCP) and contrast-enhanced CT revealed intrahepatic bile duct and proximal common bile duct dilatation, with concentric luminal narrowing of the distal intrapancreatic common bile duct, accompanied by pancreatic head enlargement without a discrete mass lesion. Imaging findings in other organs were unremarkable. Cholangiocarcinoma (CCA) was initially suspected (**Figure 1**). The patient received magnesium glycyrrhizinate and ademetionine for hepatoprotective therapy for 1 week. After 1 week of therapy, right upper-quadrant abdominal distension/pain and jaundice showed no significant improvement. Repeat complete blood count (CBC) demonstrated persistently elevated WBC count and sustained marked eosinophilia (**Table 1**, 1 week after hepatoprotective therapy).

**Table 1:** Laboratory examination results.

Test item	Result				Reference range
	Admission	1 week after hepatoprotective therapy	1 month after postoperative glucocorticoid therapy	3 months after postoperative glucocorticoid therapy	
WBC (×10^9^/L)	8.1	9	7.1	6.3	3.5-9.5
Eo# (×10^9^/L)	2.41	2.54	0.08	0.04	0.02-0.52
EO%	26.3	28	0.7	0.3	0.4-8.0
ALB (g/L)	32	28.7	34	42	40-55
ALT (U/L)	223	219	181	24	9-50
ALP (U/L)	751	697	208	83	45-125
AST (U/L)	190	234	93.6	26	13-35
TBIL (μmol/L)	182.7	161	25	12	0-21
DBIL (μmol/L)	141	91.3	15.5	6	0-6.8
IBIL (μmol/L)	41.7	69.7	9.5	6	0-26
GGT (U/L)	970	872	341	45	10-60
TBA (μmol/L)	15	20	9.2	8.1	0-15

WBC, white blood cell count; Eo#, absolute eosinophil count; EO%, eosinophil percentage; ALB, albumin; ALT, alanine aminotransferase; ALP, alkaline phosphatase; AST, aspartate aminotransferase; TBIL, total bilirubin; DBIL, direct bilirubin; IBIL, indirect bilirubin; GGT, γ-glutamyl transferase; TBA, total bile acid.

The patient had progressive obstructive jaundice with markedly elevated liver function indices (TBIL 182.7 μmol/L, DBIL 141 μmol/L), which showed no improvement after one week of hepatoprotective therapy. In addition, the patient and their family declined ERCP/EUS. Delayed surgical intervention would increase the risk of severe cholestatic liver injury and cholangitis. After multidisciplinary team discussion, the patient was diagnosed with biliary obstruction with initial suspicion for cholangiocarcinoma and was scheduled for laparoscopic pancreaticoduodenectomy. Intraoperative exploration revealed gallbladder enlargement and wall thickening secondary to biliary obstruction, but no intrinsic lesions, stones, or necrotic changes were identified. Cholecystectomy was not performed in this patient. The mid-to-distal common bile duct showed obvious induration. The pancreatic edema and pancreatic induration were also observed. These intraoperative findings favored an inflammatory biliary disease. A definitive confirmation of malignancy could not be made; therefore, a tissue diagnosis was prioritized. During the operation, we promptly communicated with the patient’s family, informed them of the intraoperative findings, and planned to terminate the pancreaticoduodenectomy and perform only bile duct exploration and drainage. The patient’s family expressed understanding and consent. Intraoperative cholangioscopy was performed and revealed smooth concentric stenosis at the distal common bile duct, with no visible mass lesion, mucosal irregularity or other malignant morphological features. Targeted bile duct wall biopsies were obtained from the mid-bile duct for histopathological diagnosis. A T-tube was placed in the mid common bile duct for biliary decompression.

**Figure 2:**
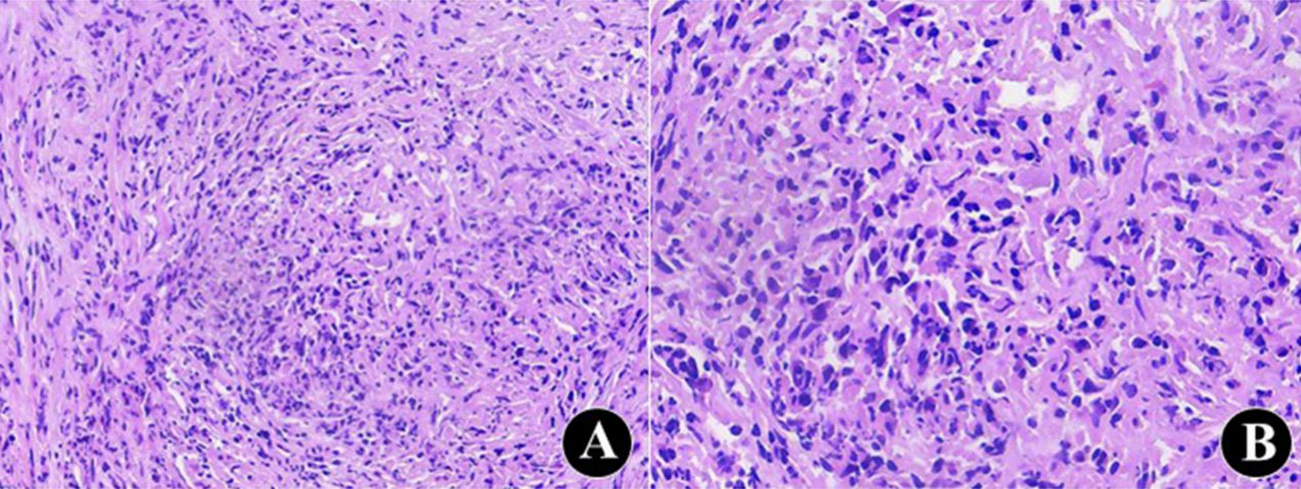
Tissue source: Targeted biopsy specimens obtained from the mid portion of the common bile duct. Hematoxylin and eosin (H&E) staining. Histopathological examination showing marked eosinophilic infiltration of the bile duct wall with eosinophilic abscess formation **(A)**; high-power magnification demonstrating diffuse eosinophilic infiltration **(B)**.

**Figure 3:**
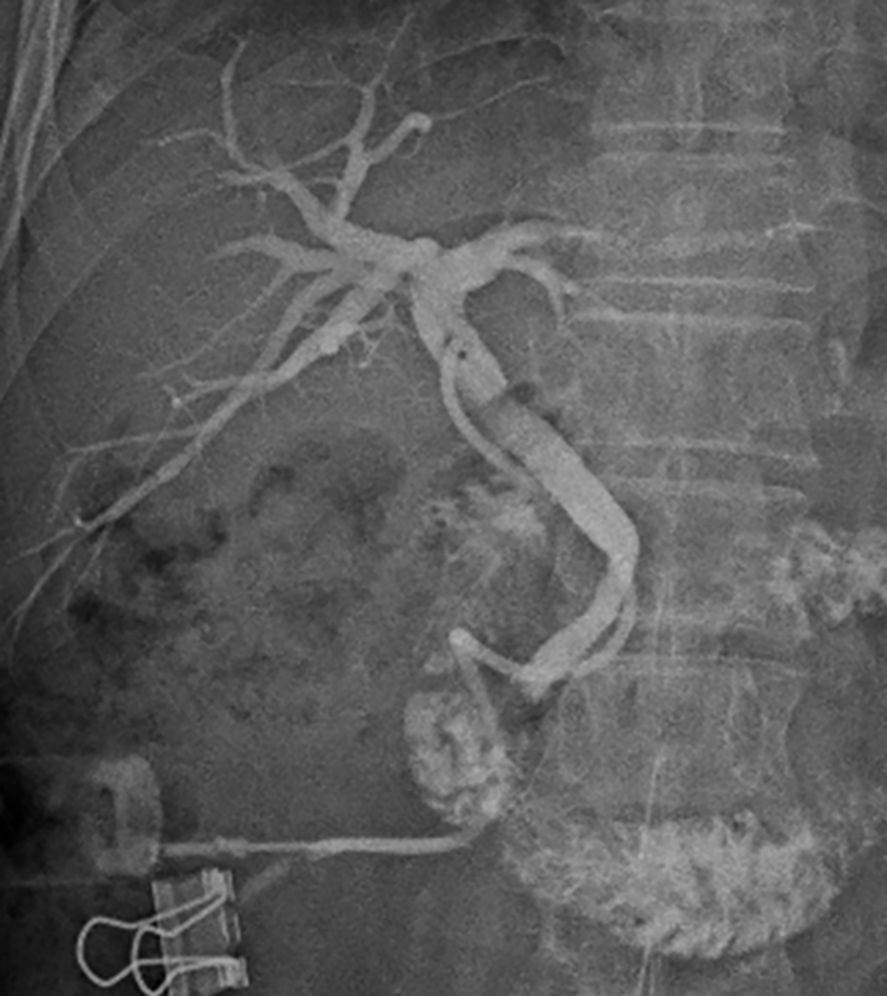
T-tube cholangiography using iodinated contrast medium performed three months after glucocorticoid therapy, demonstrating a patent bile duct.

Postoperative pathological findings showed: 1. Chronic cholangitis was observed with extensive mucosal erosion. No atypical or malignant cells were identified in the bile duct epithelium. Submucosal fibrous tissue hyperplasia was present, with abundant eosinophilic infiltration in the bile duct wall. 2. Five random high-power fields (40×) were selected from the pathological section. The mean eosinophil count exceeded 20 cells/HPF, confirming dense eosinophilic infiltration. (**Figure 2**). Notably, IgG4 immunostaining was not performed for the biliary tissue obtained by biopsy, as the preliminary pathological examination revealed marked eosinophilic infiltration and rare plasma cells in the biliary wall, with no morphological features suggestive of IgG4-related disease (IgG4-RD), such as abundant plasma cell infiltration, obliterative phlebitis, or storiform fibrosis. Based on postoperative pathology, a repeat MDT discussion was held involving rheumatology-immunology, pathology, and radiology. The patient was ultimately diagnosed with EC, obstructive jaundice, and cholestatic liver injury. A steroid treatment strategy was developed as follows: Methylprednisolone was initiated at 40 mg/day orally. The dose was tapered by 4 mg/week after 10 days, and a maintenance dose of 4 mg/day was continued from 3 months onward. During steroid therapy, calcium and vitamin D supplementation were co-administered for osteoporosis prophylaxis. Liver biochemistry was closely monitored throughout treatment. At 1- and 3-month follow-up after steroid therapy, epigastric pain and obstructive jaundice improved markedly. Serum bilirubin and transaminase levels approached the normal range and the absolute count of peripheral blood eosinophils also returned to the normal range (**Table 1**). At 3 months postoperatively, T-tube cholangiography using iohexol contrast showed patent bile ducts without residual stenosis (**Figure 3**). The T-tube was removed following imaging confirmation. The patient was followed up for 6 months after surgery and initiation of corticosteroid therapy. During this period, clinical symptoms, liver function, eosinophil count, and imaging findings all showed significant improvement.

## 3 Discussion

Eosinophilic cholangitis (EC) was first described by Leegaard in 1980 (3). EC is a rare benign inflammatory biliary disease characterized by dense eosinophilic infiltration of the bile duct wall (4), with a low clinical incidence. To date, only approximately 40 cases of EC have been reported in the literature, and its pathophysiological mechanisms remain incompletely understood. A hallmark feature of EC is bile duct wall thickening leading to biliary obstruction (5), which frequently results in misdiagnosis as cholangiocarcinoma.

Clinically, sclerosing cholangitis is classified into two categories: primary sclerosing cholangitis (PSC) and secondary sclerosing cholangitis (SSC) (6, 7). Morphologically, SSC resembles PSC. However, it is associated with well-defined pathological processes, including IgG4-related sclerosing cholangitis, EC, and intraductal bile stone disease. Notably, EC is recognized as one of the etiological causes of SSC, characterized by prominent eosinophilic infiltration of the biliary system (8). Previous studies have suggested that EC may be associated with parasitic infections, local eosinophilic inflammatory responses related to connective tissue diseases or immunodeficiencies, or allergic reactions to immunogenic stimuli(**Table 2**). Most patients with EC exhibit marked peripheral blood eosinophilia. Approximately 10% of cases involve both the bile ducts and the gallbladder (9, 10).

**Table 2:** Differential diagnosis and exclusion workup for peripheral eosinophilia.

Potential etiology of eosinophilia	Examination items	Clinical results	Reference range	Exclusion conclusion
Parasitic infection	Stool ova and parasite (O&P) examination	Negative	No parasites/ova detected	Ruled out
Allergic disease	Total serum IgE; Detailed allergy history	39 IU/mL; No allergic disease history	0–125 IU/mL	Ruled out
Drug-induced eosinophilia	Detailed medication history inquiry	No recent new drug use or hepatotoxic drug exposure	—	Ruled out
Hypereosinophilic syndrome	Chest CT; Cardiac echocardiography; Abdominal ultrasound	Imaging findings in other organs were unremarkable.	—	Ruled out
Systemic eosinophilic disorders	Systemic organ screening; Hematology consultation	No abnormal eosinophilic infiltration in systemic organs;	—	Ruled out
IgG4-related sclerosing cholangitis	Serum IgG4; Bile duct wall IgG4 immunostaining	Serum IgG4: negative; Not performed (scant plasma cells; no IgG4-related features)	IgG4-positive plasma cells: ≥10/HPF (diagnostic threshold)	Ruled out

HPF, high-power field (40×); CT, computed tomography; —, no applicable reference range.

Several studies have proposed diagnostic criteria for EC, which include thickening or stenosis of the bile duct wall, histopathological evidence of increased eosinophilic infiltration, and marked symptomatic improvement following steroid therapy (2). In laboratory examinations, elevated peripheral blood eosinophils (count >1.5×10^9^/L or proportion >10%) and increased total IgE are important indicators (1). MRCP can delineate the extent of biliary strictures and help exclude benign causes of obstruction, such as gallstones (11). Endoscopic retrograde cholangiopancreatography (ERCP) not only allows direct visualization of the biliary mucosa but also enables tissue sampling for histopathological diagnosis (12). Pathological examination remains the gold standard for confirming EC: eosinophilic infiltration in the bile duct wall (>20 cells/high-power field [HPF]). The pathological findings in this case met the aforementioned criteria, thereby establishing a definitive diagnosis of EC. Although serum IgG4 was negative in this patient, it should be emphasized that the sole use of serum IgG4 level to exclude IgG4-RD has certain clinical limitations. Serum IgG4 elevation is not a specific indicator for IgG4-RD, and a considerable proportion of IgG4-RD patients may present with normal serum IgG4 levels; in addition, mild elevation of serum IgG4 can also be seen in other inflammatory diseases, infectious diseases and malignant tumors (13). Therefore, the diagnosis or exclusion of IgG4-RD should be based on the combination of clinical manifestations, radiological features, pathological morphological characteristics and IgG4 immunostaining results, rather than a single serum IgG4 index (14). In this case, the absence of typical morphological features of IgG4-RD in biliary tissue pathology was the key reason for not performing IgG4 immunostaining, which was consistent with the clinical diagnostic logic of IgG4-RD.

Distinguishing benign from malignant biliary strictures remains a major clinical challenge in clinical practice, as their clinical manifestations and radiological features are often indistinguishable from those of cholangiocarcinoma. Although most biliary strictures are secondary to malignant tumors such as pancreatic cancer and cholangiocarcinoma, approximately 30% of cases are attributed to benign etiologies (15). Tumor markers may aid in evaluating the etiology of biliary obstruction, yet their limited specificity generally precludes definitive identification of the underlying cause (16). In the present EC case, the patient showed abnormally elevated preoperative carbohydrate antigen 19-9(CA19-9) levels, further complicating differentiation between benign and malignant biliary strictures. Peripheral eosinophilia serves as a supportive diagnostic clue for EC, however, this finding lacks disease specificity. Peripheral eosinophilia and bile duct wall thickening are also frequently observed in patients with primary sclerosing cholangitis (PSC) and IgG4-related sclerosing cholangitis (IgG4-SC) (17). Therefore, histopathological examination of biliary tissue obtained by biopsy remains essential for differential diagnosis and definitive confirmation of EC.

It should be emphasized that preoperative tissue sampling via ERCP/EUS or cholangioscopy is the principle first choice for the etiological diagnosis of biliary strictures (18), which is consistent with the gold standard status of pathological examination for EC diagnosis. Endoscopic ultrasound plays a critical role in the evaluation of periampullary lesions, including: High-resolution visualization of the ampulla, distal bile duct, and pancreatic head and differentiation of benign from malignant strictures; Accurate T and N staging for suspected malignant lesions; Guidance for fine-needle aspiration biopsy to improve preoperative pathological diagnosis (19). We acknowledge that the lack of preoperative EUS represents a minor limitation of this case, which may have restricted the more comprehensive preoperative assessment of the periampullary region and the acquisition of more precise pathological diagnostic information for this patient. However, individual clinical decision-making for preoperative tissue acquisition must be comprehensively based on practical factors including clinical urgency of the patient’s condition, the patient’s informed choice, technical feasibility of sampling, and local clinical resource conditions. For patients with progressive obstructive jaundice and unstable liver function, laparoscopic exploration with intra-operative biopsy is a reasonable and safe alternative, which can avoid the risk of delayed diagnosis and treatment caused by inconclusive preoperative sampling and reduce the potential medical risks of repeated invasive examinations.

Cholangioscopy plays a vital role in differentiating indeterminate biliary strictures suspicious for malignancy, allowing direct mucosal visualization and targeted biopsy to improve diagnostic accuracy. In this case, intraoperative cholangioscopy revealed no malignant features, suggesting an inflammatory etiology; subsequent guided biopsy confirmed eosinophilic cholangitis and avoided unnecessary radical resection.

To summarize the core causes of misdiagnosis in this case, three major factors can be identified. First, the rarity of eosinophilic cholangitis (EC) results in limited clinical awareness and insufficient diagnostic experience among clinicians, leading to misclassification as more common causes of biliary obstruction. Second, the similarity of radiological features significantly contributes to diagnostic difficulty. MRCP findings of cholangiocarcinoma typically include biliary wall thickening, biliary stricture, or dilatation. On MRCP, cholangiocarcinoma typically presents with bile duct wall thickening, biliary strictures, and proximal ductal dilatation. Similarly, EC may exhibit comparable radiologic changes secondary to bile duct wall edema and fibrosis, thereby further complicating differential diagnosis. Third, insufficient emphasis was placed on key laboratory findings. In this case, the proportion of peripheral blood eosinophils reached 28% (normal range: 0.4%–8%). In contrast, peripheral blood eosinophil counts are typically within the normal range in patients with cholangiocarcinoma.

A key aspect of the management of this case was the timely adjustment of the surgical strategy. Pancreaticoduodenectomy was initially planned preoperatively. However, intraoperative exploration revealed marked inflammatory changes involving the bile duct, pancreas, and surrounding tissues. Rather than proceeding with radical resection, the surgical plan was revised to bile duct exploration with choledochoscopic biopsy. This decision avoided unnecessary surgery associated with substantial surgical trauma and high risk. Postoperative histopathological findings established the diagnosis of EC and provided a reliable basis for subsequent glucocorticoid therapy. On the basis of the present case, we recommend that clinicians make timely adjustments to the surgical strategy when confronted with diagnostic or therapeutic dilemmas in the course of biliary surgical interventions. In addition, effective communication and informed discussion with the patient’s family are essential, which plays a critical role in minimizing medical risk.

The therapeutic strategies for eosinophilic cholangitis (EC) and cholangiocarcinoma differ fundamentally. Radical surgical resection remains the cornerstone of treatment for cholangiocarcinoma, whereas systemic glucocorticoids constitute the first-line therapy for EC with an initial dosage of 0.5–1 mg/(kg·d), followed by gradual tapering after clinical symptom improvement. In this case, jaundice resolved within one month of treatment initiation. Peripheral eosinophil counts and liver biochemistry normalized by three months, demonstrating the effectiveness of glucocorticoid therapy. It should be noted that long-term oral glucocorticoid therapy in patients with EC carries a risk of adrenal insufficiency and secondary osteoporosis. Accordingly, concomitant supplementation with calcium, vitamin D, and gastric mucosal protectants is recommended, together with regular monitoring of blood pressure, bone mineral density, and serum electrolytes. Regarding the risk of recurrence following steroid withdrawal in eosinophilic cholangitis, recurrence has been reported in approximately 20-30% of cases, primarily attributed to rapid steroid tapering, persistent eosinophilia, or underlying immune disorders (20). Therefore, long-term steroid maintenance with gradual tapering is recommended to reduce recurrence, and close long-term follow-up is essential (21).

In summary, we report a case of eosinophilic cholangitis. During the clinical management of this case, the initially planned pancreaticoduodenectomy was promptly aborted. Meticulous histopathological examination of the resected biliary tissue enabled adjustment of the postoperative treatment strategy, thereby avoiding overtreatment resulting from misdiagnosis.

## 4 Conclusion

EC should be carefully considered during the differential diagnosis of obstructive jaundice. The diagnostic and therapeutic experience from this case provides valuable insights for the management of similar biliary diseases.

## Data Availability

The datasets presented in this article are not readily available because of ethical and privacy restrictions. Requests to access the datasets should be directed to the corresponding author.
